# Effects of Endocannabinoid System Modulation on Cholinergic-Induced Anxiety-like Behaviors in Mice

**DOI:** 10.3390/ph19101574

**Published:** 2026-10-04

**Authors:** Marta Kruk-Slomka, Tomasz Slomka, Agnieszka Dzik, Grazyna Biala

**Affiliations:** 1 Department of Pharmacology and Pharmacodynamics, Medical University of Lublin, 4a Chodzki Str., 20-093 Lublin, Poland; agnieszkadzik2016@gmail.com (A.D.); grazyna.biala@umlub.pl (G.B.); 2 Experimental Medicine Center (OMD), Medical University of Lublin, Jaczewskiego 8D, 20-090 Lublin, Poland; 3 Department of Medical Informatics and Statistics with E-Health Lab, Medical University of Lublin, Jaczewskiego 4 Street, 20-954 Lublin, Poland; tomasz.slomka@umlub.edu.pl

**Keywords:** cholinergic pathways, anxiety, endocannabinoid system modulation, elevated plus maze test, mice

## Abstract

**Background:** Anxiety disorders are clinically significant non-cognitive symptoms of many neuropsychiatric disorders, often accompanying progressive memory deficits and reflecting neurochemical dysregulation, particularly cholinergic dysfunction. Current therapy for these disorders is focused on the memory deficits but does not improve emotional symptoms and may even worsen anxiety. Therefore, novel therapeutic strategies are needed that address both cognitive and non-cognitive symptoms. **Aim:** Recent studies suggest that the endocannabinoid system (ECS) may play a key role in regulating cognitive and emotional processes, including anxiety. The aim of the study was to evaluate how direct (via selective ligands of cannabinoid CB1 and CB2 receptors) and indirect (via inhibitors of endocannabinoid-degrading enzymes—fatty acid amide hydrolase (FAAH) and monoacylglycerol lipase (MAGL)) modulation of ECS activity affects anxiety-like behaviors induced by pharmacological modification of cholinergic neurotransmission (by using nicotine or scopolamine). **Methods:** Anxiety-related behaviors were assessed in mice using the elevated plus maze (EPM) test. **Results:** CB1 receptor agonist oleamide (5 mg/kg), FAAH inhibitor URB 597 (0.1 mg/kg), and MAGL inhibitor JZL 184 (20 mg/kg) attenuated the anxiogenic effects of nicotine (0.1 mg/kg). Additionally, oleamide (5 mg/kg), CB1/CB2 receptor agonist WIN 55,212-2 (0.25 mg/kg), CB2 receptor antagonist AM 630 (0.25 mg/kg), and JZL 184 (20 mg/kg) attenuated the anxiogenic effects induced by scopolamine (1 mg/kg). **Conclusions:** Both direct and indirect ECS modulation demonstrated an impact on anxiety-like responses induced by cholinergic ligands. This potential relationship between ECS and cholinergic pathways provides additional insights into anxiety regulation and may represent a promising starting point for further research into targeted anxiolytic mechanisms in the central nervous system (CNS).

## 1. Introduction

Many disorders of the central nervous system (CNS) result from irreversible damage to neuronal cells, ultimately leading to their degeneration and death. At present, these conditions are increasingly recognized as civilization-related diseases. They are particularly difficult to treat due to their highly complex pathomechanism of the diseases and the associated diverse clinical manifestations.

Anxiety disorders are clinically significant symptoms of many neuropsychiatric conditions and frequently co-occur with progressive cognitive impairment. One example of such diseases is Alzheimer’s disease (AD)—a progressive and commonly diagnosed neurodegenerative disorder, in the course of which not only cognitive decline and memory impairment are observed, but also behavioral changes and emotional disturbances such as depression and anxiety disorders. Emotional symptoms often emerge in the early stages of AD and significantly affect both treatment efficacy and patients’ quality of life [1,2,3].

Cholinergic system dysfunction plays a key role in the development of AD symptoms; in particular, a reduction in acetylcholine (ACh) level, being a consequence of the neurodegenerative process. Importantly, an increasing body of evidence suggests that anxiety symptoms in AD are not solely secondary to cognitive decline but may arise directly from neurodegenerative processes and disturbances within neurotransmitter systems. Damage to brain structures such as the prefrontal cortex and hippocampus, as well as relationships between the cholinergic system and other neurotransmitter systems, including the GABAergic, glutamatergic, and endocannabinoid systems (ECS), may further modulate the severity of anxiety symptoms [4,5].

Considerable interest in the regulation of emotional processes is currently focused on the ECS. The ECS constitutes a key neuromodulatory signaling system involved in the regulation of numerous physiological processes and CNS functions, including stress responses, learning and memory, sleep, appetite, pain perception, immune function, and anxiety-related emotional regulation. The broad involvement of the ECS in maintaining homeostasis reflects the complexity and coordinated interplay of its molecular components. The ECS comprises cannabinoid receptors (CB1 and CB2), endogenous lipid mediators known as endocannabinoids, and the enzymes responsible for their synthesis and degradation. The two main endocannabinoids, anandamide (AEA) and 2-arachidonoylglycerol (2-AG), are derived from arachidonic acid-containing membrane phospholipids and exert their effects primarily through G protein-coupled CB receptors. AEA is predominantly associated with the modulation of mood, emotional behavior, and reward-related processes, whereas 2-AG, which is present in the brain at higher concentrations, participates in a broader range of functions, including the regulation of immune responses and inflammatory processes. Endocannabinoid signaling is terminated through enzymatic degradation, mainly by fatty acid amide hydrolase (FAAH) in the case of AEA and monoacylglycerol lipase (MAGL) for 2-AG [6,7].

Recent scientific studies have focused on elucidating the role of the ECS in the regulation of emotional processes, particularly anxiety, primarily through the use of CB receptor ligands. Although the involvement of direct modulation of ECS function via CB receptor ligands in anxiety regulation has been extensively investigated, an increasing body of evidence suggests that indirect modulation of ECS function may also influence emotional behavior. Consequently, growing attention is being directed toward indirect strategies aimed at enhancing endocannabinoid signaling by inhibiting enzymes responsible for endocannabinoid degradation, such as FAAH and MAGL. Inhibition of these enzymes leads to increased levels of AEA and/or 2-AG, thereby prolonging endocannabinoid signaling. This approach is considered promising in the development of novel therapies for emotional disorders, particularly through its relationship with neuroinflammatory processes and neurotransmitter systems [8,9].

The present studies constitute a direct continuation and conceptual extension of our previous work [10], in which we investigated the effects of both indirect and direct modulation of ECS activity on anxiety-like behavior in mice using the elevated plus maze (EPM) paradigm. Those results indicated that pharmacological manipulation of ECS function could modulate anxiety-like responses. Building on those findings, and considering the known significance of cholinergic signaling and the ECS in the regulation of emotional processing, a further characterization of how these two systems may jointly influence behavior appears warranted. Therefore, based on our previously published data [10], we extended the research framework by evaluating—for the first time in our experimental model—the effects of modulating ECS activity on anxiety-like behavior induced by pharmacological modification of cholinergic neurotransmission in mice.

The aim of the present study was to determine the effects of direct and indirect modulation of ECS function on anxiety-like behavior in the context of its potential interplay with the cholinergic system. In animal experiments, the effects of CB receptor ligands as well as FAAH and MAGL inhibitors on anxiety-like behavior were assessed following acute administration of nicotine, a cholinergic receptor agonist, and scopolamine, a muscarinic receptor antagonist. Anxiety-related effects in mice were evaluated using a standard behavioral assay—the EPM test—widely employed in anxiety research and enabling the assessment of multiple dimensions of emotional behavior depending on the applied pharmacological intervention.

This study provides preliminary insights into the potential pharmacological relationship between the endocannabinoid and cholinergic systems in the regulation of anxiety in this animal model. The obtained results are discussed in the context of how direct and indirect pharmacological modulation of the ECS might modulate anxiety-like behaviors associated with cholinergic pathways. Due to their pharmacological properties and influence on emotional processing, compounds that modulate ECS function—both directly and indirectly—may represent promising research targets for further investigation into anxiolytic strategies, particularly in conditions hypothesized to involve cholinergic dysfunction and neurodegenerative processes.

## 2. Results

### 2.1. The Effects of an Acute Administration of Cholinergic Receptor Ligands on the Anxiety-Related Behavior in Mice in the EPM Test

#### 2.1.1. The Influence of an Acute Administration of Cholinergic Receptor Agonist—Nicotine on the Anxiety-Related Behavior in Mice in the EPM Test

Statistical analysis of the results using the one-way ANOVA revealed that an acute administration of nicotine at doses of 0.1 mg/kg, 0.5 mg/kg, and 1 mg/kg caused statistically significant differences between the experimental groups, both in terms of the time spent by the animals in the open arms of the EPM [F(3.32) = 5.873; *p* = 0.0029) and the number of entries into these arms [F(3.32) = 4.438; *p* = 0.0110].

**Figure 1:**
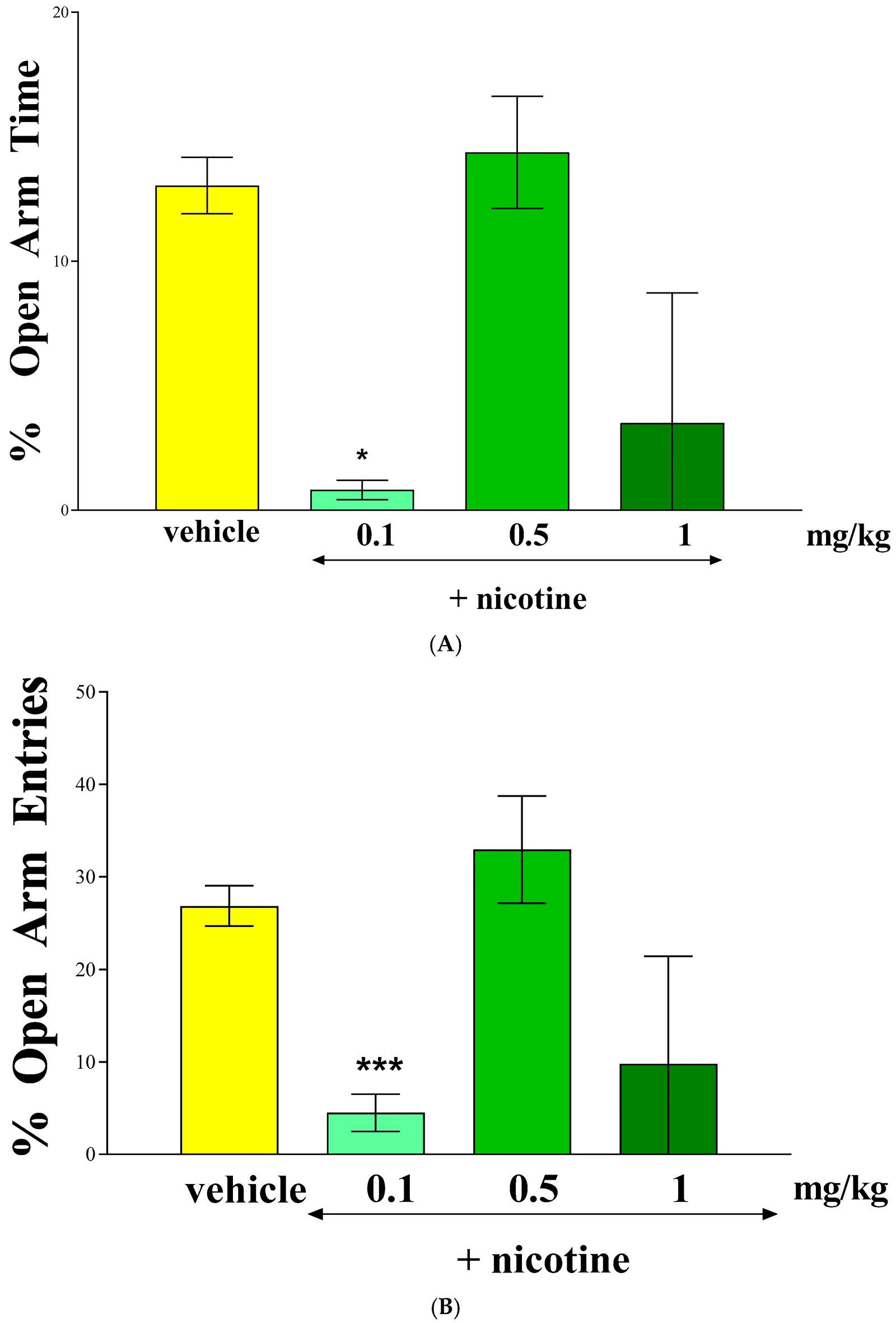
The influence of an acute administration of a cholinergic receptor agonist—nicotine—on anxiety-related behavior in mice in the elevated plus maze (EPM) test. Mean (±SEM) percentage of time spent in open arms (**A**) and percentage of open arm entries (**B**) in the EPM. * *p* < 0.05; *** *p* < 0.001 vs. vehicle-treated mice; *n* = 7–9.

Post hoc analysis using Tukey’s post hoc test confirmed a statistically significant reduction in time spent in the open arms of the EPM (*p* < 0.05) and a reduction in the number of entries into these arms (*p* < 0.001) in mice receiving nicotine injections at a dose of 0.1 mg/kg compared to the vehicle-treated control group [Figure 1A,B].

Number of closed-arm entries in the EPM: The locomotor activity of mice, expressed as the number of entries into the closed arms of the EPM, did not differ significantly between the above-mentioned experimental groups (one-way ANOVA, F(3.31) = 2.135; *p* = 0.1182) [Table 1].

#### 2.1.2. The Influence of an Acute Administration of Cholinergic Receptor Antagonist—Scopolamine on the Anxiety-Related Behavior in Mice in the EPM Test

Statistical analysis of the results using one-way ANOVA revealed that an acute injection of scopolamine at doses of 0.1 mg/kg, 0.3 mg/kg, and 1 mg/kg resulted in statistically significant differences between the experimental groups, both in terms of the time spent by the animals in the open arms of the EPM [F(3.31) = 15.68; *p* < 0.0001) and the number of entries into these arms [F(3.31) = 10.22; *p* = 0.0001].

**Figure 2:**
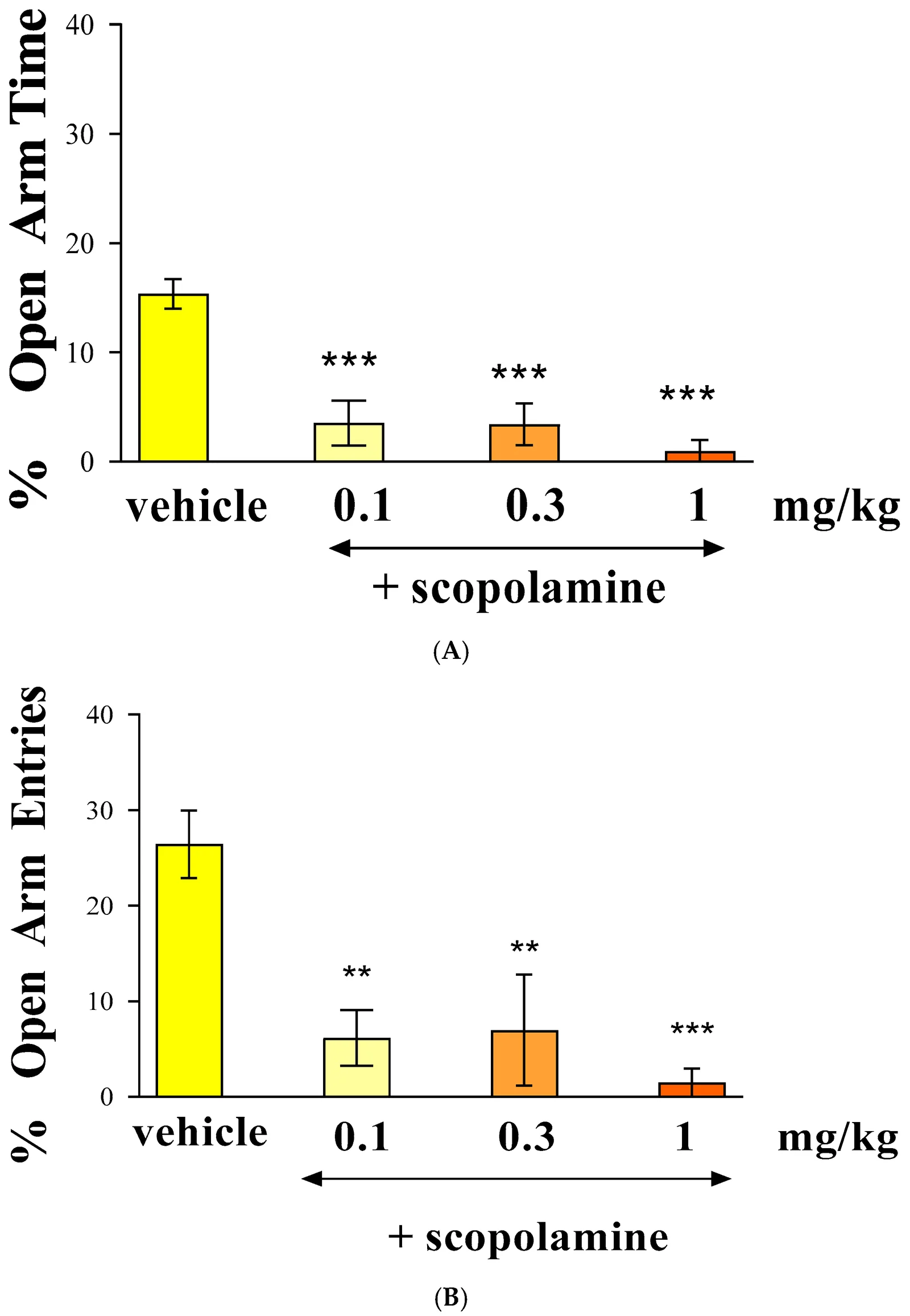
The influence of an acute administration of a cholinergic receptor antagonist—scopolamine—on the anxiety-related behavior in mice in the elevated plus maze (EPM) test. Mean (±SEM) percentage of time spent in open arms (**A**) and percentage of open arm entries (**B**) in the EPM. ** *p* < 0.01; *** *p* < 0.001 vs. vehicle-treated mice; *n* = 7–9.

Post hoc analysis using Tukey’s post hoc test showed that an acute injection of scopolamine at doses of 0.1 mg/kg, 0.3 mg/kg, and 1 mg/kg resulted in a statistically significant reduction in the time spent in the open arms of the EPM (*p* < 0.001) [Figure 2A]. Furthermore, an acute administration of scopolamine at the doses mentioned above statistically significantly reduced the number of entries into the open arms of the EPM (*p* < 0.01 for doses of 0.1 mg/kg and 0.3 mg/kg, and *p* < 0.001 for the 1 mg/kg dose), compared to the vehicle-treated control group [Figure 2B].

Number of entries into the closed arms of the EPM: The locomotor activity of mice, expressed as the number of entries into the closed arms of the EPM, did not differ statistically significantly in the above-mentioned experimental groups (one-way ANOVA, F(3.31) = 0.1822; *p* = 0.9076) [Table 2].

### 2.2. The Effects of an Acute Administration of Compounds Acting Directly on the ECS (CB Receptor Ligands) on the Anxiety-Related Responses Induced by an Acute Injection of Cholinergic Receptor Ligands (Nicotine or Scopolamine) in Mice in the EPM Test

#### 2.2.1. The Influence of an Acute Administration of Selective CB1 Receptor Ligands on the Anxiogenic Effects Induced by an Acute Injection of Nicotine in Mice in the EPM Test

Statistical analysis of the results using one-way ANOVA indicated that combined acute administration of CB1 receptor agonist, oleamide (5 mg/kg); CB1 receptor antagonist, AM 251 (3 mg/kg); or mixed CB1/CB2 receptor agonist, WIN 55,212-2 (0.25 mg/kg) together with nicotine at an effective (anxiogenic) dose of 0.1 mg/kg resulted in statistically significant differences between experimental groups, only in the terms of the number of entries into the open arms [F(3,31) = 3.484; *p* = 0.0288], but not in terms of the time spent in the open arms of the EPM [F(3,31) = 2.898; *p* = 0.0526].

**Figure 3:**
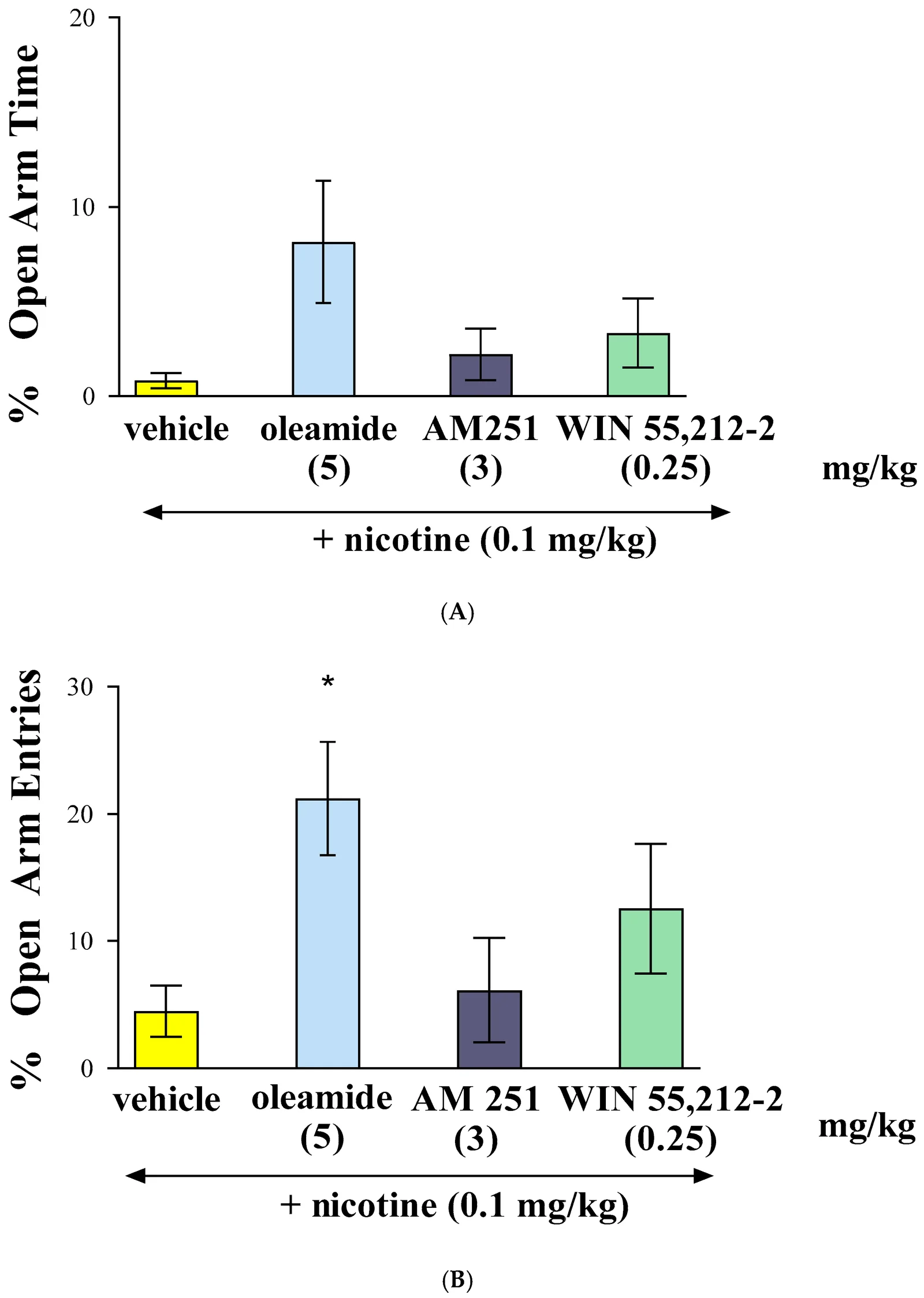
The influence of an acute administration of selective cannabinoid (CB1) receptor ligands on the anxiogenic effects induced by an acute injection of nicotine in mice in the elevated plus maze (EPM) test. Mean (±SEM) percentage of time spent in open arms (**A**) and percentage of open arm entries (**B**) in the EPM. * *p* < 0.05 vs. vehicle/nicotine (0.1 mg/kg)-treated mice; *n* = 7–9.

Post hoc analysis using Tukey’s test confirmed that an acute injection of oleamide at the dose of 5 mg/kg attenuated the anxiogenic effects induced by an acute injection of nicotine (0.1 mg/kg). This effect was reflected by a statistically significant increase in the number of entries into the open arms of the EPM (*p* < 0.05) [Figure 3B] in comparison to the control group (vehicle/nicotine (0.1 mg/kg)-treated mice). None of the remaining CB1 receptor ligands—AM 251 (3 mg/kg) and WIN 55,212-2 (0.25 mg/kg)—significantly affected either the time spent in the open arms of the EPM or the number of entries into these arms in relation to the control group.

Number of closed-arm entries in the EPM: The locomotor activity of mice, expressed by the number of entries into the closed arms of the EPM, did not differ significantly between the above-mentioned experimental groups (one-way ANOVA, F(3,31) = 0.2905; *p* = 0.8319) [Table 3].

#### 2.2.2. The Influence of an Acute Administration of Selective CB2 Receptor Ligands on the Anxiogenic Effects Induced by an Acute Injection of Nicotine in Mice in the EPM Test

Statistical analysis of the results using one-way ANOVA indicated that combined acute administration of CB2 receptor agonist—JWH 133 (0.5 mg/kg)—or CB2 receptor antagonist—AM 630 (0.25 mg/kg)—together with nicotine at an effective (anxiogenic) dose of 0.1 mg/kg did not produce any statistically significant differences between the experimental groups, either in the time spent in the open arms of the EPM [F(2,24) = 0.2125; *p* = 0.8108] or in the number of entries into the open arms [F(2,24) = 0.2124; *p* = 0.8079].

**Figure 4:**
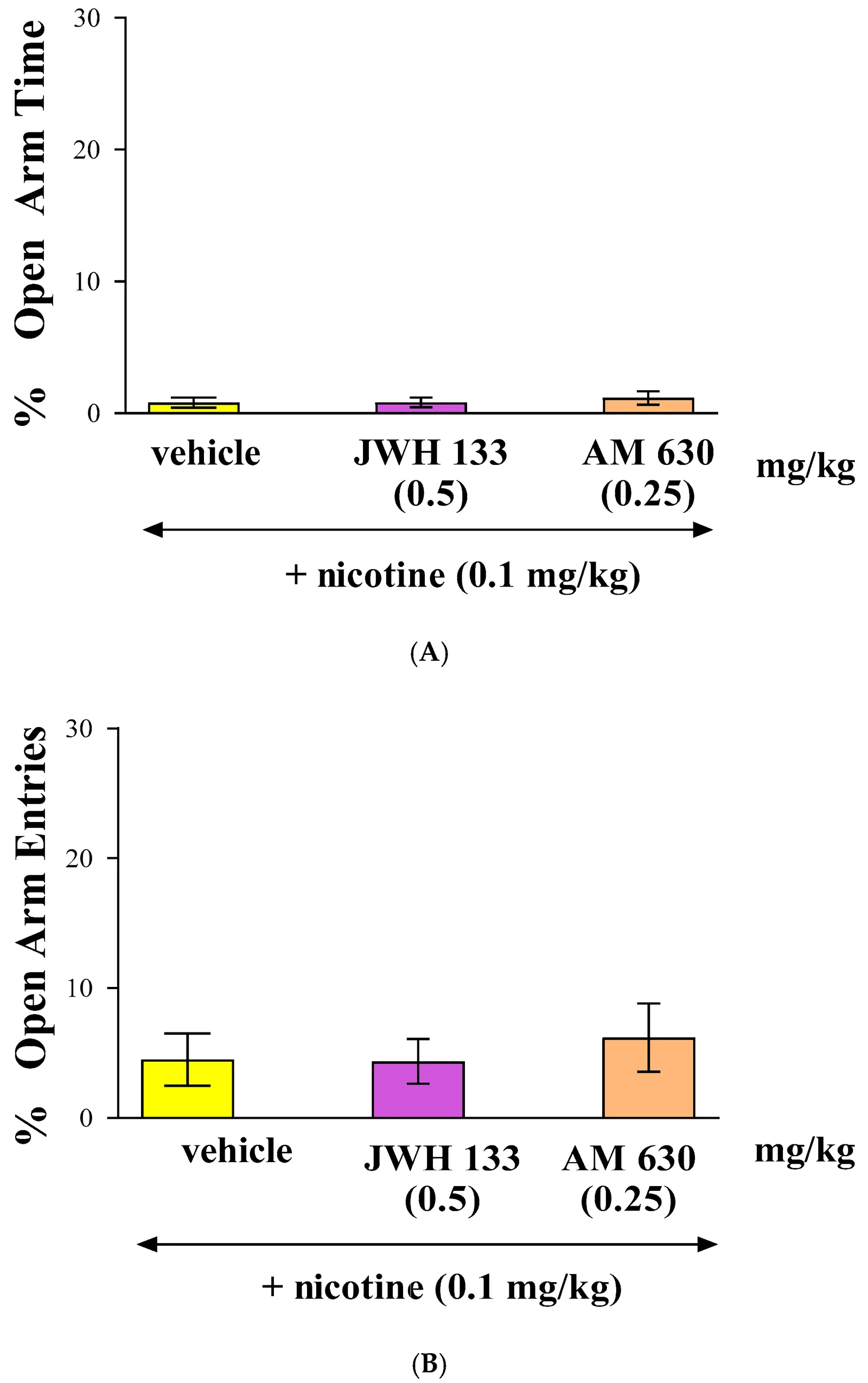
The influence of an acute administration of selective cannabinoid (CB2) receptor ligands on the anxiogenic effects induced by an acute injection of nicotine in mice in the elevated plus maze (EPM) test. Mean (±SEM) percentage of time spent in open arms (**A**) and percentage of open arm entries (**B**) in the EPM, *n* = 8–9.

Post hoc analysis using Tukey’s test confirmed that an acute injection of JWH 133 (0.5 mg/kg) and AM 630 (0.25 mg/kg) did not significantly affect nicotine-induced anxiety-like behavior following administration of nicotine at a dose of 0.1 mg/kg. This lack of effect was reflected by no significant changes in either the time spent in the open arms of the EPM [Figure 4A] or the number of entries into the open arms [Figure 4B] in comparison to the control group (vehicle/nicotine (0.1 mg/kg)-treated mice).

Number of closed-arm entries in the EPM: The locomotor activity of mice, expressed by the number of entries into the closed arms of the EPM, did not differ significantly between the above-mentioned experimental groups (one-way ANOVA, F(2,24) = 0.3042; *p* = 0.7408) [Table 4].

#### 2.2.3. The Influence of an Acute Administration of Selective CB1 Receptor Ligands on the Anxiogenic Effects Induced by an Acute Injection of Scopolamine in Mice in the EPM Test

Statistical analysis using one-way ANOVA indicated that an acute co-administration of CB1 receptor agonist, oleamide (5 mg/kg); CB1 receptor antagonist, AM 251 (3 mg/kg); or CB1/CB2 mixed receptor agonist, WIN 55,212-2 (0.25 mg/kg) together with scopolamine at an effective (anxiogenic) dose of 1 mg/kg resulted in statistically significant differences between the experimental groups, both in the time spent in the open arms of the EPM [F(3,32) = 8.499; *p* = 0.0003] and in the number of entries into the open arms [F(3,32) = 10.49; *p* < 0.0001].

**Figure 5:**
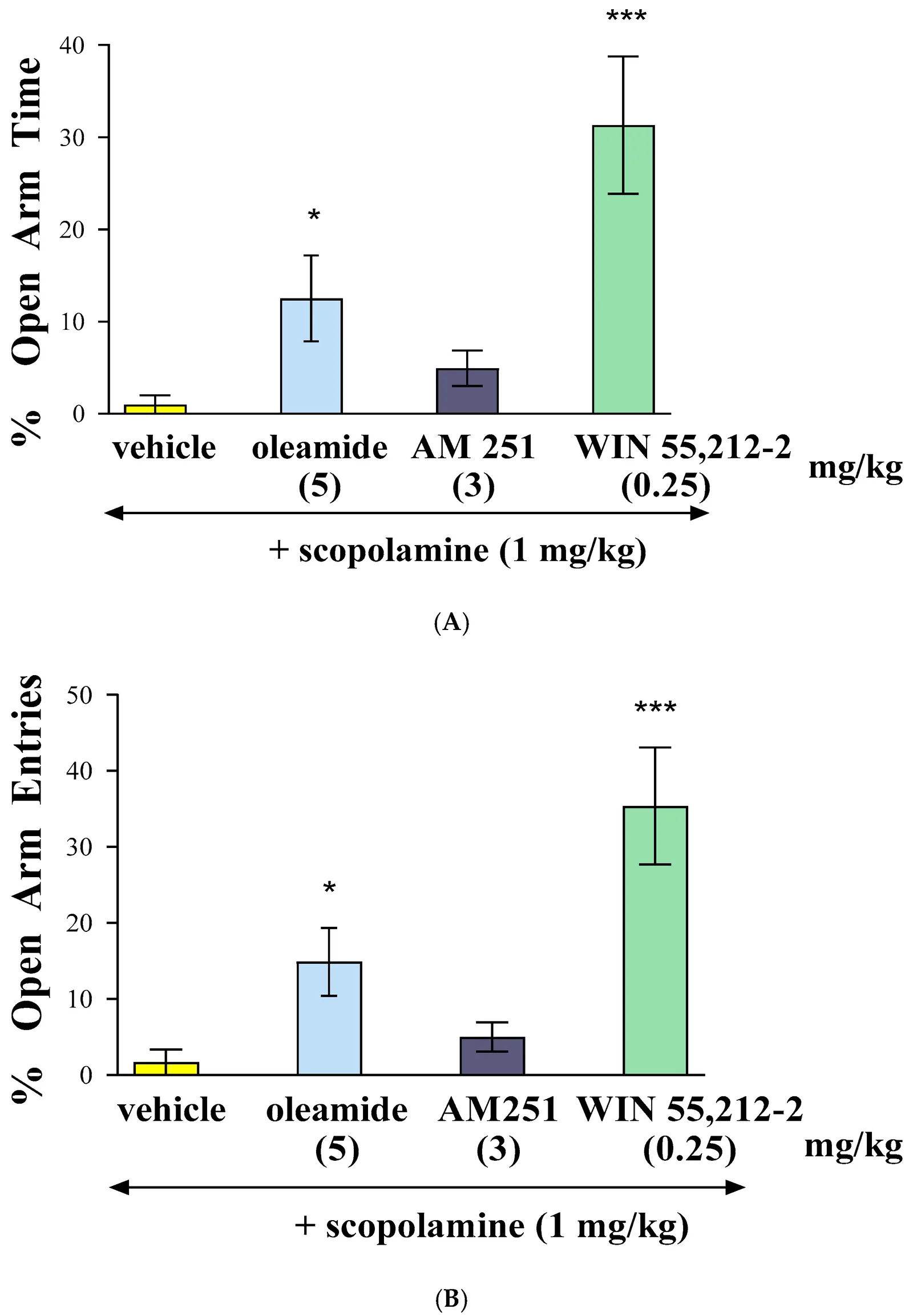
The influence of an acute administration of selective cannabinoid (CB1) receptor ligands on the anxiogenic effects induced by an acute injection of scopolamine in mice in the elevated plus maze (EPM) test. Mean (±SEM) percentage of time spent in open arms (**A**) and percentage of open arm entries (**B**) in the EPM. * *p* < 0.05; *** *p* < 0.001 vs. vehicle/scopolamine (1 mg/kg)-treated mice; *n* = 8–9.

Post hoc analysis using Tukey’s test confirmed that an acute injection of oleamide at a dose of 5 mg/kg reversed the anxiogenic effects induced by an acute injection of scopolamine. This effect was reflected by a statistically significant increase in the time spent in the open arms of the EPM (*p* < 0.05) [Figure 5A] as well as a statistically significant increase in the number of entries into the open arms (*p* < 0.05) [Figure 5B] in relation to the control group (vehicle/scopolamine (1 mg/kg)-treated mice).

Additionally, Tukey’s post hoc test confirmed that WIN 55,212-2 administered at a dose of 0.25 mg/kg also reversed the anxiogenic effects induced by scopolamine (1 mg/kg). This effect was evidenced by a statistically significant increase in both the time spent in the open arms of the EPM (*p* < 0.001) [Figure 5A] and the number of entries into the open arms (*p* < 0.001) [Figure 5B] in relation to the control group (vehicle/scopolamine(1 mg/kg)-treated mice).

Number of closed-arm entries in the EPM: The locomotor activity of mice, expressed as the number of entries into the closed arms of the EPM, did not differ significantly between the above-mentioned experimental groups (one-way ANOVA, F(3,32) = 0.007847; *p* = 0.9990) [Table 5].

#### 2.2.4. The Influence of an Acute Administration of Selective CB2 Receptor Ligands on the Anxiogenic Effects Induced by an Acute Injection of Scopolamine in Mice in the EPM Test

Statistical analysis using one-way ANOVA indicated that an acute co-administration of CB2 receptor agonist—JWH 133 (0.5 mg/kg)—or CB2 receptor antagonist—AM 630 (0.25 mg/kg)—together with scopolamine at an effective (anxiogenic) dose of 1 mg/kg resulted in statistically significant differences between the experimental groups, both in the time spent in the open arms of the EPM [F(2,22) = 4.395; *p* = 0.0262] and in the number of entries into the open arms [F(2,22) = 6.340; *p* = 0.0074].

**Figure 6:**
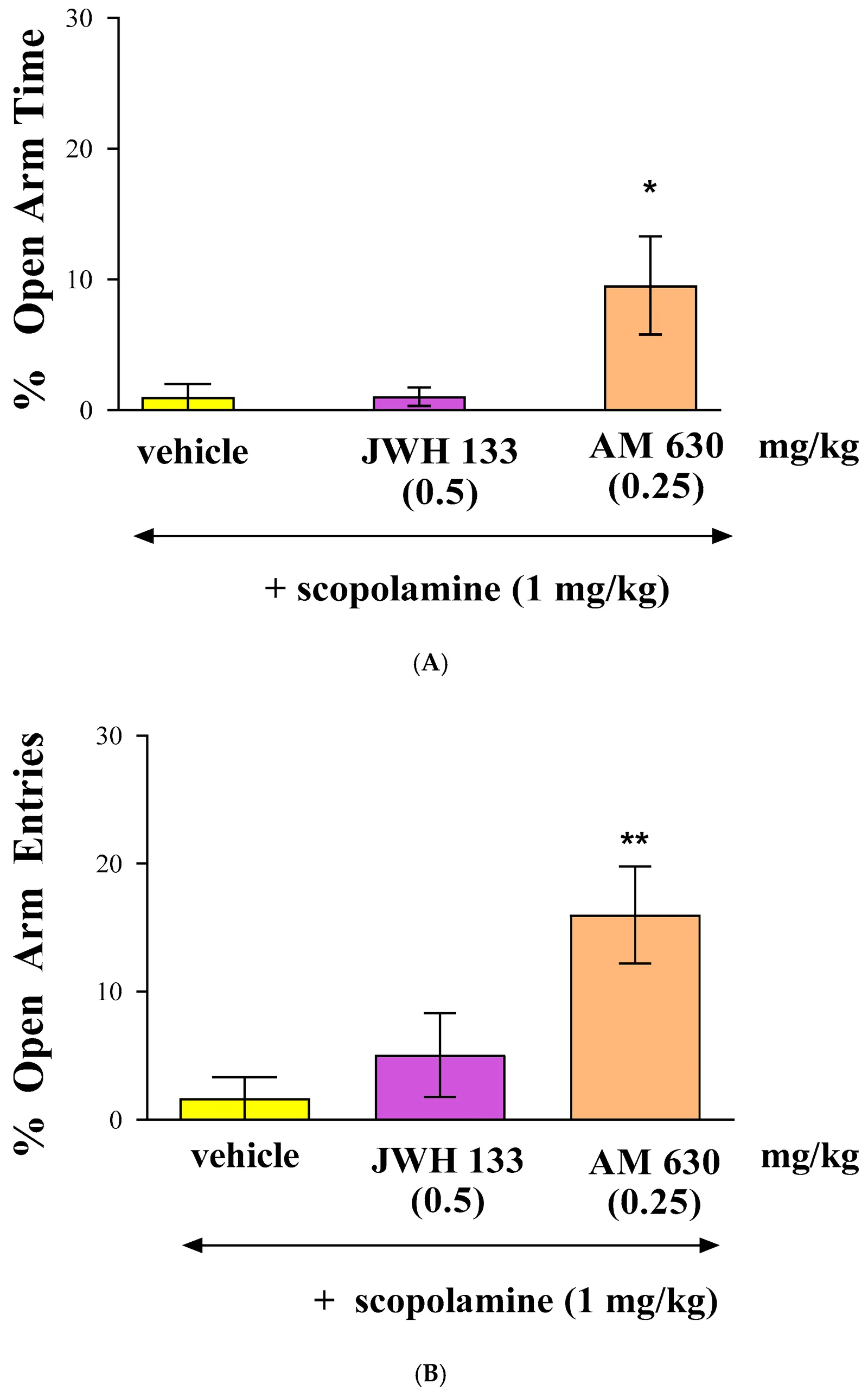
The influence of an acute administration of selective cannabinoid (CB2) receptor ligands on the anxiogenic effects induced by an acute injection of scopolamine in mice in the elevated plus maze (EPM) test. Mean (±SEM) percentage of time spent in open arms (**A**) and percentage of open arm entries (**B**) in the EPM. * *p* < 0.05, ** *p* < 0.01; vs. vehicle/scopolamine (1 mg/kg)-treated mice; *n* = 7–8.

Post hoc analysis using Tukey’s test revealed that an acute injection of AM 630 administered at a dose of 0.25 mg/kg reversed the anxiogenic effects induced by an acute injection of scopolamine. This effect was reflected by a statistically significant increase in the time spent in the open arms of the EPM (*p* < 0.05) [Figure 6A] as well as a statistically significant increase in the number of entries into the open arms (*p* < 0.01) [Figure 6B] in comparison to the control group (vehicle/scopolamine (1 mg/kg)-treated mice).

Number of closed-arm entries in the EPM: The locomotor activity of mice, expressed as the number of entries into the closed arms of the EPM, did not differ significantly between the above-mentioned experimental groups (one-way ANOVA, F(2,22) = 1.283; *p* = 0.2991) [Table 6].

### 2.3. The Effects of an Acute Administration of Compounds Acting Indirectly on the ECS (Inhibitors of Enzymes Degrading Endocannabinoids in the Brain) on the Anxiety-Related Responses Induced by an Acute Injection of Cholinergic Receptor Ligands (Nicotine or Scopolamine) in Mice in the EPM Test

#### 2.3.1. The Influence of an Acute Administration of Selective FAAH or MAGL Inhibitors on the Anxiogenic Effects Induced by an Acute Injection of Nicotine in Mice in the EPM Test

Statistical analysis using one-way ANOVA indicated that combined acute administration of FAAH inhibitor—URB 597 (0.1 mg/kg)—or MAGL inhibitor—JZL 184 (20 mg/kg)—together with nicotine at an effective (anxiogenic) dose of 0.1 mg/kg resulted in statistically significant differences between the experimental groups, both in the time spent in the open arms of the EPM [F(2,24) = 6.985; *p* = 0.0045] and in the number of entries into the open arms [F(2,24) = 6.407; *p* = 0.0064].

**Figure 7:**
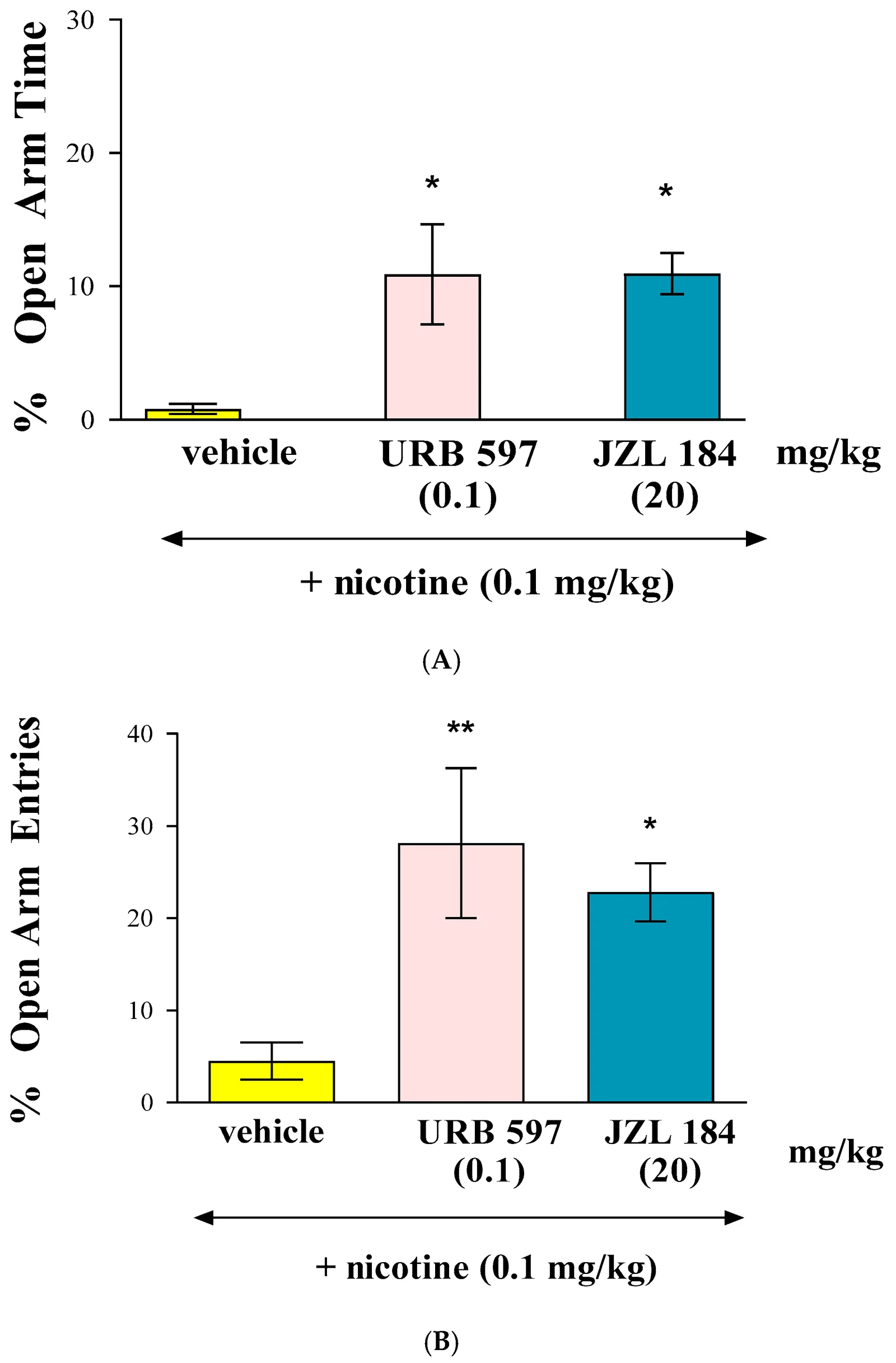
The influence of an acute administration of selective fatty acid amide hydrolase (FAAH) or monoacylglycerol lipase (MAGL) inhibitors on the anxiogenic effects induced by an acute injection of nicotine in mice in the elevated plus maze (EPM) test. Mean (±SEM) percentage of time spent in open arms (**A**) and percentage of open arm entries (**B**) in the EPM. * *p* < 0.05; ** *p* < 0.001 vs. vehicle/nicotine (0.1 mg/kg)-treated mice, *n* = 8–9.

Post hoc analysis using Tukey’s test confirmed that an acute injection of both URB 597 (0.1 mg/kg) and JZL 184 (20 mg/kg) attenuated the anxiogenic effects induced by nicotine administered at a dose of 0.1 mg/kg. This effect was reflected by a statistically significant increase in the time spent in the open arms of the EPM (*p* < 0.05) [Figure 7A]. In addition, both URB 597 (*p* < 0.01) and JZL 184 (*p* < 0.05) produced a statistically significant increase in the number of entries into the open arms of the EPM [Figure 7B] in relation to the control group (vehicle/nicotine (0.1 mg/kg)-treated mice).

Number of closed-arm entries in the EPM:The locomotor activity of mice, expressed as the number of entries into the closed arms of the EPM, did not differ significantly between the above-mentioned experimental groups (one-way ANOVA, F(2,24) = 0.2475; *p* = 0.7829) [Table 7].

#### 2.3.2. The Influence of an Acute Administration of Selective FAAH or MAGL Inhibitors on the Anxiogenic Effects Induced by an Acute Injection of Scopolamine in Mice in the EPM Test

Statistical analysis using one-way ANOVA indicated that an acute co-administration of FAAH inhibitor—URB 597 (0.1 mg/kg)—or MAGL inhibitor—JZL 184 (20 mg/kg)—together with scopolamine at an effective (anxiogenic) dose of 1 mg/kg resulted in statistically significant differences between the experimental groups, both in the time spent in the open arms of the EPM [F(2,23) = 4.721; *p* = 0.0203] and in the number of entries into the open arms [F(2,23) = 7.259; *p* = 0.0040].

**Figure 8:**
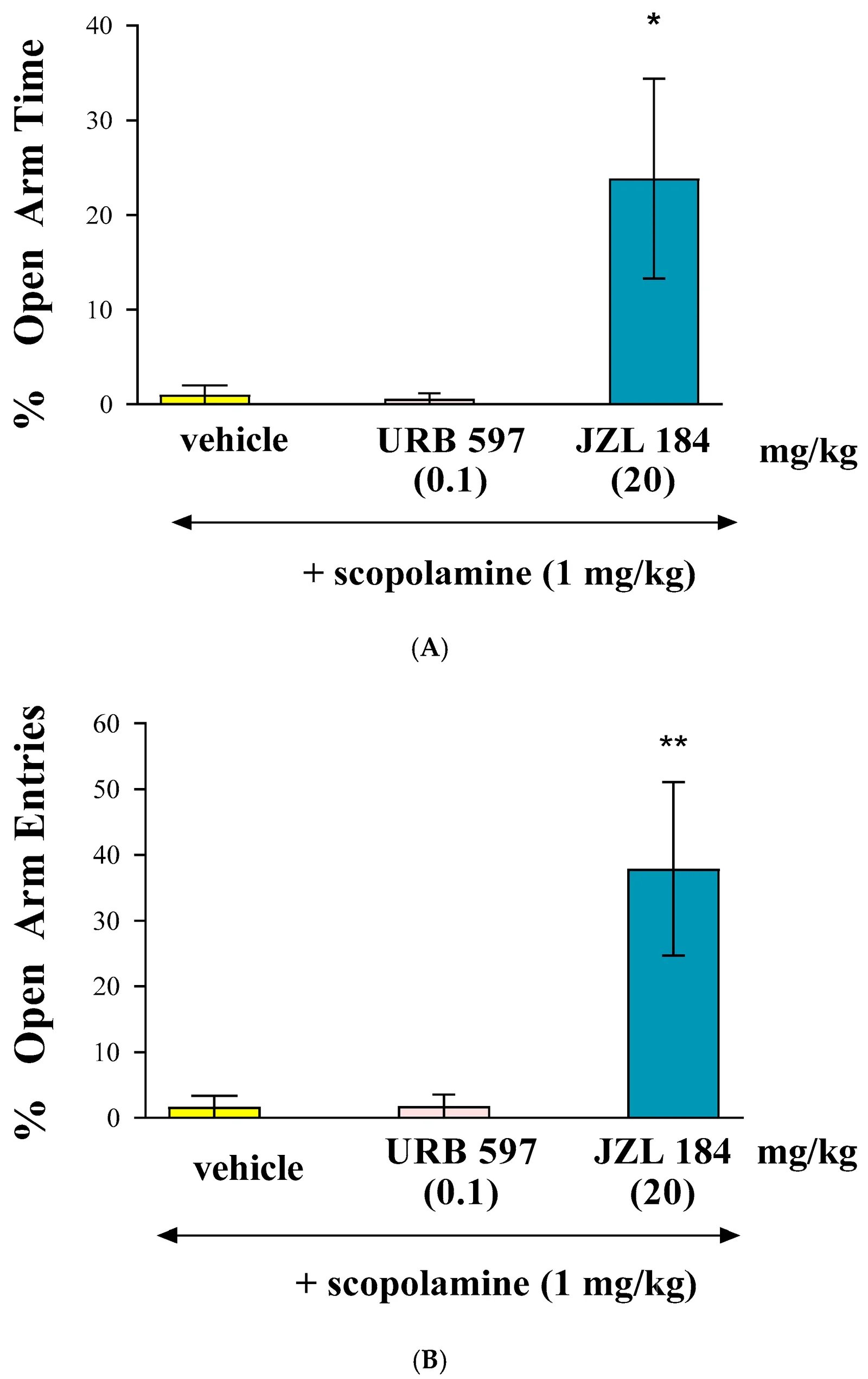
The influence of an acute administration of selective fatty acid amide hydrolase (FAAH) or monoacylglycerol lipase (MAGL) inhibitors on the anxiogenic effects induced by an acute injection of scopolamine in mice in the elevated plus maze (EPM) test. Mean (±SEM) percentage of time spent in open arms (**A**) and percentage of open arm entries (**B**) in the EPM. * *p* < 0.05; ** *p* < 0.001 vs. vehicle/scopolamine (1 mg/kg)-treated mice, *n* = 8.

Post hoc analysis using Tukey’s test confirmed that an acute injection of JZL 184 administered at a dose of 20 mg/kg reversed the anxiogenic effects induced by an acute injection of scopolamine (1 mg/kg). This effect was reflected by a statistically significant increase in the time spent in the open arms of the EPM (*p* < 0.05) [Figure 8A] as well as a statistically significant increase in the number of entries into the open arms (*p* < 0.01) [Figure 8B] in comparison to the control group (vehicle/scopolamine (1 mg/kg)-treated mice).

Number of closed-arm entries in the EPM:The locomotor activity of mice, expressed by the number of entries into the closed arms of the EPM, did not differ significantly between the above-mentioned experimental groups (one-way ANOVA, F(2,23) = 0.6195; *p* = 0.5478) [Table 8].

## 3. Discussion

Anxiety disorders constitute clinically significant non-cognitive symptoms of neurodegenerative disease, frequently co-occurring with progressive memory deficits and reflecting neurochemical dysregulation, particularly cholinergic dysfunction. Current therapeutic strategies in these disorders, including AD, primarily focus on alleviating memory impairments, provide limited improvement in emotional symptoms, and may even exacerbate anxiety. Therefore, there is an urgent need to develop novel therapeutic strategies targeting both cognitive and non-cognitive symptoms. One potential direction focuses on the ECS, a key neuromodulatory system regulating cognitive, emotional, and stress-related functions, which are often affected by changes in cholinergic signaling in central nervous system disorders [11]. Although the impact of the ECS on memory processes is well documented [12,13,14], its role in anxiety-related behaviors remains incompletely understood.

Numerous studies [15,16,17,18], including our previous work [10], suggested that the ECS can bidirectionally modulate anxiety-related behaviors, producing both anxiolytic and anxiogenic effects. Moreover, both direct and indirect pharmacological modulation of the ECS may influence anxiety responses via distinct mechanisms. Direct modulation through activation or blockade of CB1 and/or CB2 receptors, as well as indirect modulation via inhibition of endocannabinoid-degrading enzymes (FAAH and MAGL), may represent a promising target for further research into therapeutic strategies. Our previous studies [10] suggested that the CB1 receptor agonist oleamide did not affect anxiety-related responses in mice in the EPM test, whereas the CB1 receptor antagonist AM 251 induced an anxiogenic effect. Moreover, acute administration of the mixed CB1/CB2 receptor agonist WIN 55,212-2 produced an anxiolytic effect. Regarding CB2 receptors, acute administration of the CB2 agonist JWH 133, as well as the CB2 antagonist AM 630, induced anxiogenic effects. In turn, an acute administration of the FAAH inhibitor, URB 597, produced dose-dependent anxiolytic effects, whereas the MAGL inhibitor, JZL 184, did not significantly affect anxiety-related behaviors [10]. Based on these previous findings, we designed subsequent studies to further evaluate the behavioral effects of ECS-modulating compounds in the context of anxiety-like responses that model pathological conditions, such as cholinergic dysfunction. Since limited studies have focused on the relationship between the ECS and cholinergic systems in the context of anxiety-like behavior, our experiments were planned to explore how modulation of the ECS could influence these cholinergic-induced phenotypes. This approach is consistent with the growing body of research suggesting that a complex interplay among multiple neurotransmitter systems may underlie the regulation of anxiety states [19].

Similar to our previous work [10], in the present study, anxiety-like behaviors in mice were assessed using the EPM test based on established anxiety-related parameters: percentage of entries into open arms, percentage of time spent in open arms, and the number of entries into closed arms as a measure of locomotor activity. Taking into account the assumptions described above, the specific aim of the present study was to evaluate the effects of direct ECS modulation (using selective CB1 and CB2 receptor ligands) as well as indirect ECS modulation (via inhibitors of endocannabinoid-degrading enzymes—FAAH and MAGL) on anxiety-like behaviors induced by pharmacological disruption of cholinergic neurotransmission. To assess these relationships between both systems, two cholinergic receptor ligands were selected—nicotine and scopolamine—which may induce and modulate anxiety-related responses [20,21,22,23,24]. Our results suggest a nonlinear, biphasic effect of nicotine on anxiety-related behavior in the EPM test. In the present study, a single administration of nicotine at the lowest dose (0.1 mg/kg) produced an anxiogenic-like effect, which reached statistical significance. A similar direction of effect was observed at the highest dose (1 mg/kg), although the difference did not reach statistical significance. In contrast, the intermediate dose of nicotine (0.5 mg/kg) produced an anxiolytic-like effect, although this effect was also not statistically significant. This response profile further supports the concept that the influence of nicotine on anxiety-related behavior is not linear and may depend strictly on the dose administered. The present findings are partially consistent with previous observations indicating a complex dose–response relationship between nicotine and anxiety-related behavior. Biala and Budzynska [21] reported that acute administration of nicotine at a dose of 0.1 mg/kg s.c. produced an anxiogenic-like effect in mice in the EPM. However, in contrast to our findings, the 0.5 mg/kg dose also produced an anxiogenic-like effect in their study. On the other hand, available evidence suggests that the effects of nicotine may follow a biphasic pattern. Balerio et al. (2006) [22] indicated in mice that a low dose of nicotine (0.05 mg/kg) produced an anxiolytic-like effect, whereas a higher dose (0.8 mg/kg) produced an anxiogenic-like effect in the EPM test. The effects of nicotine have also been associated with adaptations within the receptor system. Previous studies have reported that acute administration of nicotine at a dose of 0.1 mg/kg may produce an anxiogenic-like effect, whereas chronic exposure may lead to tolerance to this effect, followed by the development of an anxiolytic-like response [22,23,24].

In the present study, acute administration of nicotine (0.1 mg/kg) or scopolamine (1 mg/kg) produced pronounced anxiety-like effects in the EPM test, and on this basis, these two doses were selected for the next stage of our experiments. Such an experimental approach enabled the assessment of the effects of both direct and indirect ECS modulation on anxiety-like behaviors induced by nicotine or scopolamine, with the aim of offering preliminary insights into the potential pharmacological relationship between the ECS and the cholinergic system in the context of anxiety-like responses.

Our results suggest that co-administration of selective CB receptor ligands or ECS enzyme inhibitors with nicotine may lead to differential modulation of nicotine-induced anxiety-like effects. Specifically, acute administration of the FAAH inhibitor URB 597 (0.1 mg/kg) and the MAGL inhibitor JZL 184 (20 mg/kg) significantly attenuated the anxiogenic effects of nicotine (0.1 mg/kg), as reflected by an increased percentage of entries into open arms and prolonged time spent in the open arms in the EPM test. In the case of the CB1 receptor agonist, oleamide (5 mg/kg), we observed that this compound weakened only the percentage of entries into the EPM open arms, but had no influence on the percentage of time spent in the open arms.

In turn, the CB1/CB2 receptor agonist WIN 55,212-2 (0.25 mg/kg), the CB2 receptor agonist JWH 133 (0.5 mg/kg), or the CB2 receptor antagonist AM 630 (0.25 mg/kg) did not significantly alter EPM parameters disrupted by nicotine. Similarly, the anxiogenic effects induced by an acute scopolamine administration (1 mg/kg) were significantly suppressed by an acute administration of the CB1 receptor agonist oleamide (5 mg/kg), the mixed CB1/CB2 receptor agonist, WIN 55,212-2 (0.25 mg/kg); the CB2 receptor antagonist, AM 630 (0.25 mg/kg); and the MAGL inhibitor, JZL 184 (20 mg/kg), thereby normalizing open-arm exploration in the EPM disturbed by scopolamine. In contrast, an acute administration of the CB2 receptor antagonist, AM 251 (3 mg/kg); the CB2 receptor agonist, JWH 133 (0.5 mg/kg); or the FAAH inhibitor, URB 597 (0.1 mg/kg) did not significantly influence scopolamine-induced anxiogenic responses in mice in the EPM test.

Our findings suggest a potential role of the ECS in modulating anxiety-like behaviors associated with cholinergic disturbances, which may involve several neurobiological mechanisms related to a hypothetical interplay between the cholinergic system and the ECS. Enhanced cholinergic neurotransmission has repeatedly been associated with increased anxiety-like behaviors in rodents, leading to the literature hypotheses that excessive ACh activity may potentially promote hyperreactivity of limbic–hypothalamic circuits [25]. The ECS strongly modulates the activity of key brain regions involved in anxiety regulation, e.g., amygdala (generation of anxiety responses), hippocampus (contextual processing of fear and anxiety), and prefrontal cortex (cognitive control of emotional responses). CB1 receptors are located mainly presynaptically on glutamatergic and cholinergic nerve terminals in these structures. Presynaptically localized CB1 receptors modulate the release of multiple neurotransmitters via Gi/o protein-dependent mechanisms involving inhibition of adenylyl cyclase and N-/P-/Q-type calcium channels. Activation of CB1 receptors leads to inhibition of calcium ions (Ca^2+^) influx into presynaptic terminals, reducing the release of Glu and ACh. Following that, activation of CB1 receptors in these brain regions leads to reduced neuronal excitability and attenuation of anxiety responses. In this context, the ECS may function as a compensatory mechanism limiting excessive activation of cholinergic pathways under stress conditions [26,27]. Differences in the efficacy of ECS ligands may result from distinct mechanisms of action of nicotine and scopolamine. Concerning the pharmacological relationships between the ECS and the cholinergic system via cholinergic muscarinic receptors (mAChRs), the ECS (primarily through CB1 receptors) and the cholinergic system (via mAChRs, particularly the M1 subtype) exhibit bidirectional interactions within the CNS [28]. Specifically, activation of M1 receptors stimulates intracellular signaling pathways that lead to the production of endocannabinoids (e.g., AEA and 2-AG), which subsequently act as retrograde messengers on presynaptic CB1 receptors [29,30]. Conversely, activation of CB1 receptors located on cholinergic terminals inhibits ACh release, resulting in cholinergic hypofunction and establishing a feedback loop between these systems [31]. Both systems show strong convergence in the hippocampus and cortex, regions critically involved in learning, memory, and emotional processing [32]. Scopolamine is a competitive antagonist of mAChRs, widely employed to induce transient cholinergic hypofunction and cognitive impairment as a pharmacological model of dementia [33,34]. Under conditions of cholinergic blockade, ECS activation may compensate for the scopolamine-induced reduction in cholinergic tone. This effect may involve modulation of residual ACh release, altering network plasticity, or indirect upregulation of muscarinic signaling [30]. Experimental evidence suggests that CB1 agonists (e.g., WIN55,212-2) can prevent scopolamine-induced memory deficits by increasing cortical cholinergic activity and enhancing muscarinic receptor-related signaling in the cortex [35]. In turn, CB1 antagonists (e.g., AM281) reverse these scopolamine-induced deficits by increasing cholinergic tone [36]. Overall, these findings support the hypothesis that the ECS may act as a modulatory or compensatory regulator of cholinergic transmission. However, under pathological or pharmacologically induced cholinergic deficits (e.g., following scopolamine administration), this potential relationship becomes more dynamic and context-dependent [30]. As described in the literature, at the molecular, synaptic, and behavioral levels, M1 receptor activation appears to trigger endocannabinoid signaling, whereas CB1 receptor activation suppresses cholinergic transmission. This hypothesized interplay is particularly relevant for anxiety-related behaviors and may represent a promising target for the further investigation of therapies aimed at cognitive and affective disorders [37]. Concerning the functional relationship between the ECS and nicotinic acetylcholine receptors (nAChRs), it can be noted that these systems also interact at multiple levels, particularly in brain regions associated with reward, anxiety, cognition, and addiction, such as the hippocampus, prefrontal cortex, and ventral tegmental area (VTA) [38,39]. nAChRs, particularly the α4β2 and α7 subtypes, mediate fast excitatory neurotransmission, whereas presynaptic CB1 receptors regulate the release of multiple neurotransmitters, including dopamine, glutamate, GABA, and ACh [40]. Activation of nAChRs enhances neurotransmitter release, while CB1 receptor activation suppresses this effect, meaning the ECS acts as a counter-regulatory system to nicotinic excitation [30,41]. Nicotine, a prototypical nAChR agonist, indirectly engages the ECS; its activation leads to Ca^2+^ influx, stimulating the production of endocannabinoids (e.g., AEA and 2-AG). These molecules subsequently act as retrograde messengers on presynaptic CB1 receptors to reduce neurotransmitter release under excessive nicotinic activation. Accordingly, CB1 receptor antagonists (e.g., rimonabant) have been shown to reduce nicotine self-administration and decrease relapse-like behavior, whereas CB1 receptor agonists enhance the reinforcing effects of nicotine. As for the nAChRs-ESC interplay mentioned above, this suggests that the ECS provides a homeostatic feedback mechanism that constrains and shapes nicotine-induced neurobehavioral effects [42]. In summary, ECS–nAChR interactions occur via Ca^2+^-dependent endocannabinoid release and CB1 receptor signaling. Nicotine indirectly activates the ECS within reward and emotional circuits, while CB1 activation dampens nicotinic transmission. Further exploration of this hypothesized interplay may contribute to a better understanding of the neurobiological mechanisms underlying addiction, cognition, and anxiety-related neuropsychiatric disorders [39,43].

Our results suggest that activation of the CB1 receptor by oleamide attenuated the anxiogenic effects of both nicotine and scopolamine. Among the CB receptor ligands used in our experiments, the pharmacological effects of oleamide warrant further in-depth analysis. Oleamide is an endogenous substance naturally occurring in both animal and human organisms. Due to its chemical structure similarity to AEA, it exhibits a broad spectrum of biological activities, including sleep induction, immunosuppressive effects, hypolocomotion, as well as antidepressant-like properties and modulation of memory and learning [44]. In the context of our experiments, data concerning the effects of oleamide on anxiety-related reactions are particularly important. However, its influence on anxiety-like behavior remains poorly documented, and the available findings are inconclusive and often contradictory. In anxiety tests such as the EPM and the light–dark box in mice, oleamide shows a tendency toward anxiolytic-like effects [10,45]. In our previous study [10], the ambiguous nature of oleamide’s effects on anxiety-related behavior was also highlighted. In those experiments, oleamide at the applied doses (5–20 mg/kg) did not produce a statistically significant effect on anxiety levels in mice in the EPM test, though a tendency toward increased open arm entries and time spent was observed. In contrast, the present work indicates that oleamide at a dose of 5 mg/kg attenuated the anxiogenic effects induced by scopolamine or partially alleviated those induced by nicotine in the EPM test in mice. The weak and often statistically non-significant anxiolytic-like effect of oleamide largely depends on the administered dose and experimental conditions. Lower and moderate doses are more frequently associated with anxiolytic-like effects, whereas higher doses may induce sedation and reduced locomotor activity, masking or modifying anxiety-like behaviors in behavioral tests. The multidirectional mechanism of action of oleamide may further contribute to this variability. Due to its structural similarity to AEA, oleamide may serve as a substrate for the FAAH enzyme, potentially enhancing the effects of other endocannabinoids by competing for enzymatic degradation. It has also been suggested that oleamide acts as a full CB1 receptor agonist, producing a range of pharmacological effects associated with its activation, including anxiety reduction [44,46]. The observed relationships between oleamide and other compounds modulating central nervous system function, such as nicotine and scopolamine, provide additional behavioral insights. Both nicotine and scopolamine act on neurotransmitter systems involved in anxiety regulation, including the cholinergic system, which is hypothesized to functionally interact with the ECS.

While oleamide exerts little or no significant effect on muscarinic cholinergic receptors, metabotropic glutamate receptors, NMDA (N-methyl-D-aspartate) receptors, or AMPA (α-amino-3-hydroxy-5-methyl-4-isoxazolepropionic acid) receptors [47], the mechanism by which it attenuates the nicotine- or scopolamine-induced anxiety remains unclear. In this context, oleamide, as a compound with cannabinoid-like properties, may indirectly modulate these effects. Preclinical studies have shown that modulation of the ECS can alter the behavioral effects of nicotine and scopolamine, including their anxiety-related components, suggesting functional interplay between these systems [20,26,48,49]. The observed effects likely result from overlapping mechanisms involving CB1 receptors, alongside the GABAergic, serotonergic, and cholinergic systems, representing simultaneous modulation of multiple neurotransmitter networks.

In turn, the mixed CB1/CB2 receptor agonist WIN 55,212-2 produced an anxiolytic effect against scopolamine-induced anxiety-like responses, while exhibiting no significant effect on nicotine-induced anxiety-like behaviors in mice. This effect is consistent with previous studies, demonstrating the influence of WIN 55,212-2 on the ACh level in selected brain regions. Tazavara et al. [30] reported that administration of this CB compound produces a biphasic, dose-dependent effect on the ACh level in the hippocampus. Namely, low doses of WIN 55,212-2 (0.5 mg/kg, i.p.) induced a transient stimulation of ACh release, while high doses of WIN55,212-2 (5 mg/kg, i.p.) caused a prolonged inhibition of ACh release via CB1 receptors coupled to Gi proteins. Other authors also reported apparently contradictory—both stimulatory and inhibitory—effects of cannabinoids on the ACh level in another brain structure: the cerebral cortex [50]. The mixed CB1/CB2 receptor agonist WIN 55,212-2 caused an anxiolytic effect only in the scopolamine-induced model. Similarly, the CB1 receptor antagonist AM 251 and selective CB2 receptor ligands (JWH 133, AM 630) indicated limited efficacy in the nicotine model but partially modulated scopolamine-induced effects, indicating that the outcome depends on the type of cholinergic disturbance (muscarinic versus nicotinic). In addition, inhibition of specific endocannabinoid-degrading enzymes (mainly metabolizing AEA or 2-AG) indicated significant differential effects on anxiety-like behaviors induced by nicotine or scopolamine in the EPM. The MAGL inhibitor JZL 184 reduced anxiety-like behaviors induced by both nicotine and scopolamine, whereas the FAAH inhibitor URB 597 was effective only in the nicotine model. These data suggest that 2-AG, as the principal endogenous agonist in the ECS, may play a more dominant role in regulating anxiety associated with cholinergic neurotransmission.

CB1-dependent presynaptic inhibition remains the primary mechanism responsible for anxiety-related responses in our experiments. Most of the available literature data indicate that ligands acting on CB2 receptors exert a less direct influence on mood and behavior compared to ligands acting on CB1 receptors. Nevertheless, CB2 receptors may indirectly modulate anxiety-related responses through immune-related mechanisms, including the regulation of inflammatory processes and a potential influence on neuroinflammation, which has been linked to the pathogenesis of anxiety disorders. The literature data regarding the role of CB2 receptors in anxiety-related behaviors are often inconsistent and inconclusive. Available findings indicate antidepressant, anxiolytic, and anxiogenic effects, as well as a lack of significant influence on anxiety-related behavior [51,52]. Our previous studies [10] also indicated that both acute administration of a CB2 receptor agonist (JWH-133 at doses of 1 and 2 mg/kg) and a CB2 receptor antagonist (AM630 at doses of 0.5–2 mg/kg) induced anxiogenic effects in the EPM test in mice. In contrast, in the present study, we revealed that only the CB2 receptor antagonist AM630 attenuates anxiogenic effects induced by nicotine and scopolamine.

The presented data suggest a complex modulatory role for CB2 receptor signaling, rather than a simple unidirectional influence on anxiety-like responses in this animal anxiety model. AM630, as a selective CB2 receptor antagonist, may modulate anxiety-related behavior in mice in a strongly context-dependent manner. In behavioral paradigms such as the EPM test, acute administration of AM630 has been described as producing anxiogenic-like effects, whereas the literature data indicate that its chronic administration may lead to opposite effects, depending on experimental conditions [51]. The bidirectional and context-dependent effects observed following pharmacological modulation with both CB2 receptor agonists and antagonists are consistent with the concept that these receptors function as state-dependent modulators of neural circuit activity rather than classical linear effectors. While the precise underlying pathways remain to be fully characterized, the literature suggests that CB2 receptor activity could influence anxiety-like behavior through indirect modulation of neuroimmune signaling and potential, speculative effects on other neurotransmitter systems, which may help provide a purely theoretical context for our behavioral observations.

Based on the obtained results, it may be hypothesized that CB2 receptor activity could be involved in the modulation of behaviors, including anxiety-like responses associated with the cholinergic system in this model. In this context, AM630 may modify the behavioral effects of nicotine as well as scopolamine. Consequently, the effects of CB2 receptor antagonism observed in our study might reflect a potential, indirect modulation of cholinergic signaling, leading to alterations in anxiety responses induced by pharmacological modulation of the cholinergic system [52]. Additionally, attention should be paid to the localization of CB2 receptors, which are predominantly expressed in microglia and other immune-related cells within the brain. Their activation is particularly relevant under conditions such as neuroinflammation, neuronal damage, and chronic stress. Consequently, in this specific context of acute cholinergic disturbance (e.g., following nicotine administration), the impact of CB2 receptor activation on behavior may be limited. In contrast, in the scopolamine-induced model, there might be a potential activation of inflammatory or oxidative stress pathways, which could help explain the observed effectiveness of specific CB2 receptor ligands in modulating behavioral outcomes [53,54].

### Limitations of Study

Several limitations of the present study should be acknowledged: 

Four-week-old mice were selected to evaluate anxiety-like behaviors in the EPM test during early adolescence, which represents a critical developmental stage for the maturation of the neural circuits underlying fear and anxiety. Furthermore, conducting investigations on animals at this pre-pubertal age allowed us to minimize the potential confounding effects of circulating sex hormones, whose capacity to modulate open-arm exploration in adult rodents is well documented. Specifically, only male mice were utilized in the present study to ensure a homogeneous baseline during the pre-pubertal period and to avoid potential confounding factors associated with the earlier and more variable onset of puberty in female rodents at this age. Moreover, it should be noted that this was a pilot study conducted exclusively on juvenile male mice to minimize initial biological variance and limit animal usage. However, when interpreting the generalizability of our findings within a broader context, the profound impact of sex-dependent dimorphism within both the endocannabinoid and cholinergic systems must be considered. In rodents, the expression and binding affinity of CB1 receptors, as well as tissue levels of endocannabinoids such as AEA and 2-AG, exhibit significant baseline differences between sexes, which are frequently modulated by fluctuating ovarian hormones. Similarly, the cholinergic system displays distinct, sex-specific profiles, particularly regarding ACh release and AChE activity within neuroanatomical regions critical for anxiety regulation, such as the hippocampus and the amygdala. Given that the interaction between endocannabinoid and cholinergic transmission is heavily modulated by GABAergic and glutamatergic systems during emotional behaviors, including anxiety, the pharmacological interactions observed in our male-only study might manifest differently in females. In particular, the onset of estrus-induced hormonal surges could alter CB1 receptor sensitivity or modify cholinergic tone, potentially shifting behavioral thresholds for anxiety-like responses in the EPM test. Consequently, while our study establishes a robust baseline in pre-pubertal males, caution is warranted when extrapolating these specific interactions to female populations, underscoring the necessity of incorporating gender-balanced cohorts in future investigations to fully map these neurochemical pathways.

The next limitation of this study is the exclusive reliance on a single behavioral paradigm, the EPM test, to characterize anxiety-like states. Although the incorporation of complementary paradigms—such as the open field or light–dark box tests—could provide a more comprehensive multidimensional behavioral profiling, the EPM was deliberately selected as a highly sensitive, specific, and independently validated conflict-based model. This focused approach was strategically employed to maintain strict methodological consistency with our previous investigation and to mitigate confounding factors associated with multi-test batteries, including cumulative stress, learning effects, and habituation. To maximize the interpretive value within this single paradigm, multiple complementary parameters were rigorously evaluated, using open-arm exploration as a primary index of anxiety and closed-arm entries as an auxiliary, context-dependent proxy for general locomotor activity. Nevertheless, we acknowledge that closed-arm entries do not represent a pure, independent measure of locomotion under high-aversive conflict conditions. Consequently, future studies are warranted to expand upon these functional behavioral outcomes by integrating distinct behavioral paradigms to fully dissociate general locomotion from specific anxiety-related phenotypes.

The final limitation of the present work is the absence of concomitant biochemical and molecular analyses to evaluate the specific neurochemical pathways involved. Consequently, and as requested, conclusions regarding the hypothesized cholinergic–endocannabinoid relationship and its precise underlying molecular mechanisms have been carefully qualified in this revised version and must be interpreted with caution. However, as an exploratory pilot trial, this work successfully establishes a robust behavioral phenotype in the EPM, providing a solid foundation for future mechanistic investigations. Therefore, expanding this work to include comprehensive biochemical and neurobiological validation to further elucidate this potential receptor interplay represents a priority for our future research directions. Moreover, future studies are warranted to fully investigate these anxiety-like phenotypes in female cohorts during the same developmental window.

## 4. Materials and Methods

### 4.1. Animals

The experiments were conducted on naïve male Swiss albino mice obtained from the Center for Experimental Medicine (Lublin, Poland), at 4 weeks of age, weighing 20–25 g. The animals were housed in groups of 10 per cage and maintained under standard laboratory conditions (12 h light/dark cycle and room temperature of 21 ± 1 °C), with free access to tap water and standard laboratory chow (Bacutil, Motycz, Poland). The mice were allowed to acclimatize to the laboratory environment for one week prior to the experiments. The animals’ housing environment was enriched with cardboard tunnels and wooden blocks to enhance welfare and promote natural behaviors. Each experimental group consisted of 7–9 animals. All behavioral tests were performed between 8:00 a.m. and 3:00 p.m., in accordance with the natural light–dark cycle. The study comprised eight independent behavioral experiments conducted in mice using the EPM test. The total number of unique animals used in the study was 226.

Animals were randomly assigned to experimental groups prior to the EPM test, with the individual animal as the experimental unit. Each mouse received treatment according to group allocation and was analyzed independently. Group sizes (*n* = 7–9 per group) were based on the prior literature and pilot data, balancing the detection of biologically meaningful effects with ethical considerations. No formal a priori power calculation was performed. Allocation sequences were generated manually by an independent blinded investigator not involved in behavioral testing to prevent bias.

To minimize potential confounders, animals from different groups were tested in alternating order, and cage positions within the housing racks were regularly rotated to control for environmental variables, including light, temperature, and handling differences.

All experimental procedures were carried out in compliance with the ARRIVE guidelines for reporting animal research and in accordance with the National Institutes of Health Guide for the Care and Use of Laboratory Animals, as well as the European Community Council Directive of 22 September 2010 (2010/63/EU). In addition, all experiments were approved by the Local Ethical Committee for Animal Experiments in Lublin (Approval Code: 13/2020; Approval Date: 24 February 2020).

The selection of male animals was based on ethical approvals and experimental design considerations aimed at reducing variability associated with the estrous cycle, which could influence both behavioral outcomes and endocannabinoid signaling.

### 4.2. Drugs

To assess interactions between the ECS and cholinergic systems, we used cholinergic receptor ligands to modulate the cholinergic system, together with compounds acting directly (CB1 or CB2 receptor agonists and antagonists) or indirectly (FAAH and MAGL inhibitors) on the ECS.

**The cholinergic receptor ligands:**

- Nicotine (0.1, 0.5, 1 mg/kg) (Tocris, Minneapolis, MN, USA), a cholinergic nicotinic receptor agonist;
- Scopolamine (0.1, 0.3, 1 mg/kg) (Tocris, USA), a cholinergic muscarinic receptor antagonist.

**The compounds acting directly on the ECS:**

- Oleamide (cis-9-octadecenamide; Tocris, USA): 5 mg/kg—CB1 receptor agonist;
- AM 251 (N-(piperidin-1-yl)-5-(4-iodophenyl)-1-(2,4-dichlorophenyl)-4-methyl-1H-pyrazole-3-carboxamide; Tocris, USA): 3 mg/kg—CB1 receptor antagonist;
- WIN 55,212-2 ([R(+)-[2,3-dihydro-5-methyl-3-[(morpholinyl)methyl]pyrrolo[1,2,3-de]-1,4-benzoxazinyl]-(1-naphthalenyl)methanone mesylate]; Tocris, USA): 0.25 mg/kg—CB1/CB2 receptor agonist;
- JWH 133 (3-(1′,1′-dimethylbutyl)-1-deoxy-Δ8-tetrahydrocannabino; Tocris, USA): 0.25, 0.5, 1, 2 mg/kg—CB2 receptor agonist;
- AM 630 ((6-iodo-2-methyl-1-[2-(morpholinyl)ethyl]-1H-indol-3-yl)(4-methoxyphenyl)methanone; Tocris, USA): 0.25, 0.5, 1, 2, 3 mg/kg—CB2 receptor antagonist.

**The compounds acting indirectly on the ECS:**

- URB 597 ([3-(3-carbamoylphenyl)phenyl] N-cyclohexylcarbamate; Tocris, USA): 0.1, 0.3, 1 mg/kg—FAAH inhibitor;
- JZL 184 (4-nitrophenyl-4-[bis(1,3-benzodioxol-5-yl)(hydroxy)methyl]piperidine-1-carboxylate; Tocris, USA): 2, 4, 8, 20, 40 mg/kg—MAGL inhibitor.

Cholinergic receptor ligands, CB receptor compounds, and inhibitors were suspended in a 1% solution of Tween 80 (Sigma, St. Louis, MO, USA) in a saline solution. Nicotine was administered subcutaneously (s.c.); scopolamine and ECS-targeting compounds were administered intraperitoneally (i.p.) at a volume of 10 mL/kg. Fresh drug solutions were prepared daily. Control animals received injections of vehicle (saline with Tween 80) at the same volume and via the same route of administration.

Experimental doses of cholinergic ligands and compounds modulating ECS function used for behavioral experiments and procedures were chosen according to those frequently used in our previous experiments [10,21].

### 4.3. Experimental Procedure

Anxiety-like behaviors in mice were evaluated using the EPM test, a well-established paradigm for assessing anxiety-related responses in rodents. The procedure followed the methodology originally described by Lister [40,41]. The EPM apparatus (obtained from ATANER, Lublin, Poland) was made of dark Plexiglas and consisted of four arms arranged in a plus configuration around a central platform measuring 5 × 5 cm. Two opposite arms were open (30 × 5 cm), whereas the other two arms were enclosed by walls (30 × 5 × 15 cm). The maze was elevated 50 cm above the floor to enhance anxiety-related behaviors. All experiments were conducted in a quiet, dimly lit room, with the central platform uniformly illuminated by soft red light.

Each mouse was placed individually on the central platform, facing one of the closed arms, and allowed to explore the maze freely for 300 s. An arm entry was recorded when all four paws crossed the boundary between the central platform and the respective arm. Because rodents naturally avoid open arms, the following parameters were assessed.

Time spent in the open arms was expressed as a percentage of the total test duration, and the number of open-arm entries was expressed as a percentage of total entries into both open and closed arms. Increases in these measures were interpreted as anxiolytic-like effects, whereas decreases reflected anxiogenic-like effects.

Number of closed-arm entries and total arm entries, used as indices of the locomotor activity during the test [55,56].

### 4.4. Treatment

#### 4.4.1. The Effects of Cholinergic Receptor Ligands

Cholinergic ligands (nicotine (0.1, 0.5, 1 mg/kg, s.c.), scopolamine (0.1, 0.3, 1 mg/kg, i.p.), or the vehicle for the control group were administered 30 min before the EPM test. Based on these experiments, we have chosen the effective dose of nicotine, 0.1 mg/kg, and the effective dose of scopolamine, 1 mg/kg [Table 9].

Next, we evaluated the influence of ECS on the anxiety-related responses induced by cholinergic receptor ligands in the EPM task.

#### 4.4.2. Nicotine-Induced Anxiogenic Effects

Compounds acting directly on the ECS:

- Oleamide (CB1 agonist, 5 mg/kg), AM 251 (CB1 antagonist, 3 mg/kg), WIN 55,212-2 (CB1/CB2 agonist, 0.25 mg/kg), JWH 133 (CB2 agonist, 0.5 mg/kg), and AM 630 (CB2 antagonist, 0.25 mg/kg).

As well as compounds acting indirectly on the ECS:

- URB 597 (FAAH inhibitor, 0.1 mg/kg) and JZL 184 (MAGL inhibitor, 20 mg/kg) were administered as single doses 15 min prior to nicotine injection (0.1 mg/kg, s.c.).

The EPM test was conducted 15 min after nicotine administration. The results were compared with vehicle-pretreated, nicotine-treated controls [Table 10].

#### 4.4.3. Scopolamine-Induced Anxiogenic Effects:

Compounds acting directly on the ECS:

- Oleamide (CB1 agonist, 5 mg/kg), AM 251 (CB1 antagonist, 3 mg/kg), WIN 55,212-2 (CB1/CB2 agonist, 0.25 mg/kg), JWH 133 (CB2 agonist, 0.5 mg/kg), and AM 630 (CB2 antagonist, 0.25 mg/kg).

As well as compounds acting indirectly on the ECS:

- URB 597 (FAAH inhibitor, 0.1 mg/kg) and JZL 184 (MAGL inhibitor, 20 mg/kg) were administered as single doses 15 min prior to scopolamine injection (1 mg/kg, i.p.).

The EPM test was performed 15 min after scopolamine administration. The results were compared with vehicle-pretreated, scopolamine-treated controls [Table 11].

### 4.5. Statistical Analysis

The statistical analyses were performed using one-way analysis of variance (ANOVA). The *post hoc* comparison of means was carried out with Tukey’s test for multiple comparisons, when appropriate. The data were considered statistically significant at a confidence level of *p* < 0.05.

The one-way ANOVA was selected due to its established robustness against departures from normality in nearly balanced experimental designs (*n* = 7–9 per group).

All statistical analyses were carried out using GraphPad Prism software (version 5.00 for Windows; GraphPad Software, San Diego, CA, USA).

No formal inclusion or exclusion criteria were predefined a priori. All animals allocated to the experimental groups that completed the study procedures were included in the analysis. Data points were excluded only in rare instances of technical issues (e.g., equipment malfunction or animal escape during behavioral testing). No animals or experimental units were excluded from the analysis for treatment-related reasons.

Any occasional exclusions were solely attributable to technical problems encountered during behavioral testing (e.g., interrupted recordings or incomplete trials). The total number of such cases did not exceed one or two per experiment and had no impact on group balance or statistical power.

## 5. Conclusions

The present findings suggest that pharmacological modulation of the endocannabinoid system can modify cholinergic-induced anxiety-like responses in mice. In the present experimental model, both direct modulation of the ECS using selective cannabinoid receptor ligands and indirect modulation through inhibition of the endocannabinoid-degrading enzymes FAAH and MAGL altered anxiety-like responses induced by pharmacological manipulation of cholinergic neurotransmission with nicotine or scopolamine. These findings indicate only that, under the specific experimental conditions used, modulation of the ECS can influence anxiety-like responses associated with cholinergic receptor stimulation or blockade in mice. The observed effects provide preliminary behavioral indications of a potential relationship between endocannabinoid and cholinergic mechanisms in the regulation of anxiety-like behavior in this animal model. However, the considerable variability observed across the experimental groups warrants strict caution in the interpretation and generalization of these findings. Such variability may be related to several factors, including animal strain and sex, experimental conditions, behavioral procedures, previous pharmacological manipulations, drug doses, and baseline anxiety levels. Overall, we merely suggest a potential functional relationship between the two systems in preclinical tests. The present findings do not establish a direct mechanistic interaction between the endocannabinoid and cholinergic systems. These results provide initial preclinical evidence that modulation of the ECS affects cholinergic-induced anxiety-like responses in mice and may serve as a starting point for further behavioral, biochemical, and molecular studies aimed at characterizing the potential mechanisms underlying these effects.

## Figures and Tables

**Table 1:** The locomotor activity of mice, expressed as the number of entries into the closed arms of the EPM, measured after an acute administration of nicotine (0.1–1 mg/kg), mean (±SEM), *n* = 7–9.

Acute Drug Injection	Mean Entries into EPM Closed Arms	Number of Mice
Vehicle (i.p.)	12.13 ± 1.27	7
Nicotine (0.1 mg/kg, s.c.)	12.33 ± 2.35	9
Nicotine (0.5 mg/kg, s.c.)	25.67 ± 9.19	8
Nicotine (1 mg/kg, s.c.)	9.75 ± 2.25	8

**Table 2:** The locomotor activity of mice, expressed as the number of entries into the closed arms of the EPM, measured after an acute administration of scopolamine (0.1–1 mg/kg), mean (±SEM), *n* = 7–9.

Acute Drug Injection	Mean Entries into EPM Closed Arms	Number of Mice
Vehicle (i.p.)	12.13 ± 1.27	7
Scopolamine (0.1 mg/kg, i.p.)	13.75 ± 2.96	8
Scopolamine (0.3 mg/kg, i.p.)	10.38 ± 5.07	9
Scopolamine (1 mg/kg, i.p.)	13.86 ± 4.45	8

**Table 3:** The locomotor activity of mice, expressed as the number of entries into the closed arms of the EPM, measured after an acute administration of the following substances (mean (±SEM), *n* = 7–9).

Acute Drug Injection		Mean Entries into EPM Closed Arms	Number of Mice
Vehicle (i.p.) + nicotine (0.1 mg/kg, s.c.)		12.33 ± 2.35	9
Oleamide (5 mg/kg, i.p.)	+nicotine (0.1 mg/kg, s.c.)	10.38 ± 2.13	7
AM 251 (3 mg/kg, i.p.)		8.88 ± 3.98	8
WIN 55,212-2 (0.25 mg/kg, i.p.)		9.13 ± 3.23	8

**Table 4:** The locomotor activity of mice, expressed as the number of entries into the closed arms of the EPM, measured after an acute administration of the following substances (mean (±SEM), *n* = 8–9).

Acute Drug Injection		Mean Entries into EPM Closed Arms	Number of Mice
Vehicle (i.p.) + nicotine (0.1 mg/kg, s.c.)		12.33 ± 2.35	9
JWH 133 (0.5 mg/kg, i.p.)	+nicotine (0.1 mg/kg, s.c.)	10.25 ± 3.41	8
AM 630 (0.25 mg/kg, i.p.)		9.13 ± 3.23	8

**Table 5:** The locomotor activity of mice expressed as the number of entries into the closed arms of the EPM, measured after acute administration of the following substances (mean (±SEM), *n* = 8–9).

Acute Drug Injection		Mean Entries into EPM Closed Arms	Number of Mice
Vehicle (i.p.) + scopolamine (1 mg/kg, i.p.)		14.97 ± 3.64	8
Oleamide (5 mg/kg, i.p.)	+scopolamine (1 mg/kg, i.p.)	14.25 ± 4.33	9
AM 251 (3 mg/kg, i.p.)		15.13 ± 9.31	8
WIN 55,212-2 (0.25 mg/kg, i.p.)		15.63 ± 7.73	8

**Table 6:** The locomotor activity of mice expressed as the number of entries into the closed arms of the EPM, measured after acute administration of the following substances (mean (±SEM), *n* = 7–8).

Acute Drug Injection		Mean Entries into EPM Closed Arms	Number of Mice
Vehicle (i.p.) + scopolamine (1 mg/kg, i.p.)		14.97 ± 3.64	8
JWH 133 (0.5 mg/kg, i.p.)	+scopolamine (1 mg/kg, i.p.)	6.88 ± 4.73	7
AM 630 (0.25 mg/kg, i.p.)		15.25 ± 3.99	8

**Table 7:** The locomotor activity of mice, expressed as the number of entries into the closed arms of the EPM, measured after an acute administration of the following substances (mean (±SEM), *n* = 8–9).

Acute Drug Injection		Mean Entries into EPM Closed Arms	Number of Mice
Vehicle (i.p.) + nicotine (0.1 mg/kg, s.c.)		12.33 ± 2.35	9
URB 597 (0.1 mg/kg, i.p.)	+nicotine (0.1 mg/kg, s.c.)	9.75 ± 3.69	8
JZL 184 (20 mg/kg, i.p.)		10.13 ± 2.42	8

**Table 8:** The locomotor activity of mice, expressed as the number of entries into the closed arms of the EPM, measured after an acute administration of the following substances (mean (±SEM), *n* = 8).

Acute Drug Injection		Mean Entries into EPM Closed Arms	Number of Mice
Vehicle (i.p.) + scopolamine (1 mg/kg, i.p.)		14.97 ± 3.64	8
URB 597 (0.1 mg/kg, i.p.)	+scopolamine (1 mg/kg, i.p.)	15.63 ± 8.43	8
JZL 184 (20 mg/kg, i.p.)		6.75 ± 5.85	8

**Table 9:** The scheme of cholinergic receptor ligands (nicotine or scopolamine) or vehicle administration during the assessment of mice behavior in the elevated plus maze (EPM) test.

Drug Administration	Interval	Parameters Assessed in the EPM Test
Cholinergic receptor ligands: Nicotine (0.1; 0.5; 1 mg/kg) Scopolamine (0.1; 0.3; 1 mg/kg)	30 min	% Open Arm Time % Open Arm Entries Number of Closed Arm Entries
vehicle	30 min	

**Table 10:** The scheme of cannabinoid (CB) receptor ligands or inhibitors of selective fatty acid amide hydrolase (FAAH) or monoacylglycerol lipase (MAGL) and nicotine co-administration during the assessment of mice behavior in the elevated plus maze (EPM) test.

Drug Administration	Interval	Drug Administration	Interval	Parameters Assessed in the EPM Test
CB receptor ligands: oleamide (5 mg/kg) AM-251 (3 mg/kg) WIN 55,212-2 (0.25 mg/kg) JWH 133 (0.5 mg/kg) AM 630 (0.25 mg/kg)	15 min	Nicotine (0.1 mg/kg)	15 min	% Open Arm Time % Open Arm Entries Number of Closed Arm Entries
Inhibitors of FAAH or MAGL: URB 597 (0.1 mg/kg) JZL 184 (20 mg/kg)	15 min	Nicotine (0.1 mg/kg)	15 min	
Vehicle (control group)	15 min	Nicotine (0.1 mg/kg)	15 min	

**Table 11:** The scheme of cannabinoid (CB) receptor ligands or inhibitors of selective fatty acid amide hydrolase (FAAH) or monoacylglycerol lipase (MAGL) and scopolamine co-administration during the assessment of anxiety-related behavior in mice in the elevated plus maze (EPM).

Drug Administration	Interval	Drug Administration	Interval	Parameters Assessed in the EPM Test
CB receptor ligands: Oleamide (5 mg/kg), AM-251 (3 mg/kg), WIN 55,212-2 (0.25 mg/kg) JWH 133 (0.5 mg/kg) AM 630 (0.25 mg/kg)	15 min	Scopolamine (1 mg/kg)	15 min	% Open Arm Time % Open Arm Entries Number of Closed Arm Entries
Inhibitors of FAAH or MAGL: URB 597 (0.1 mg/kg) JZL 184 (20 mg/kg)	15 min	Scopolamine (1 mg/kg)	15 min	
Vehicle (control group)	15 min	Scopolamine (1 mg/kg)	15 min	

## Data Availability

The original contributions presented in this study are included in the article. Further inquiries can be directed to the corresponding author.
